# Socio-economic and environmental impacts of Syrian Refugees in Jordan: A Jordanians’ perspective

**DOI:** 10.1016/j.heliyon.2022.e10005

**Published:** 2022-07-20

**Authors:** Hamzah Khawaldah, Nidal Alzboun

**Affiliations:** Geography Department, Faculty of Arts, The University of Jordan, Jordan

**Keywords:** Syrian refugees, Jordanians’ perception, Impacts, Syrian crisis, Jordan

## Abstract

Jordan has received more than 1.2 million Syrian refugees, including 656,213 registered with United Nations High Commissioner for Refugees (UNHCR) since the beginning of Syrian crisis in 2011. Jordanians are acutely feeling significant impacts of the refugees on their daily lives and refugees' pressure on services, natural resources and the labor market, especially in host communities where the impact is the highest. This study aims to analyze economic, social and environmental impacts of Syrian refugees in Jordan from Jordanians' point of view. It also aims to identify the suggestions of Jordanians to alleviate the impacts of Syrians and adapt with the refugees issue in Jordan. This study relies primarily on the available data of Syrian refugees taken from the relevant corporations in Jordan such as Department of Statistics, Ministry of Planning and International Cooperation (MOPIC), and UNHCR. In addition, a questionnaire was designed and distributed online to a representative sample of Jordanians (1080 respondents) to achieve the study’s objectives. The results revealed that pressure on labor market, services, and infrastructure as well as increasing housing rents were the main economic impacts of Syrian refugees especially in the neighboring governorates of Syria in Jordan. The social impacts were represented by increasing crime rates and drugs. Refugees also have impacts and pressure on clean water, energy and environment. However, the respondents reported some positive impacts of refugees including providing market with highly-skilled and low-waged labors that makes the Jordanian market more competitive. The study concluded with some suggested recommendations that may alleviate the negative impacts of refugees on Jordan and adapt with the crisis.

## 1 Introduction

Jordan witnessed many migration waves in the last seven decades, including Palestinian migrants in 1948 and 1967, the return of large number of Jordanian and Palestinians from gulf countries in 1991 after the gulf war in 1990, Iraqi refugees in 2003, and Syrian refugees recently. The instability in the Middle East had affected Jordan in different ways and clearly on the country’s economy. For example, Saif and DeBartolo (2007) and Glonek (2014) have studied the impact of Iraqi refugees on Jordan’s economy, showing negative impacts in certain aspects such as the increase in food prices, petroleum, and properties and some positive impacts such as new investments by Iraqi refugees.

The civil war in Syria, which started in 2011, resulted in massive wave of refugees out of Syria into many countries in the world as 5.1 million left the country and became refugees. The neighboring countries of Syria have been affected by this crisis such as Turkey, Lebanon, and Jordan (Esen and Oğuş Binatlı, 2017). In particular, Jordan has received a large number of Syrian refugees who have firstly settled in the refugees' camps in north and northeast of Jordan as well as in Amman. Following the length of the Syrian conflict, the number of Syrian refugees in Jordan increased and they started to move and live in the neighboring cities and towns affecting the local economy and hosted communities. Syrian refugees have and are still adding strain on Jordan’s economy and infrastructure, putting pressure on many sectors including education, health, housing, water, municipal services and electricity. Jordanians in general, and host communities in particular, feel the impact of refugees on their daily lives and pressure on local service delivery, natural resources and the labor market (Ministry of Planning and International Cooperation (MOPIC), 2019). Esen and Oğuş Binatlı (2017) argue that the impact of Syrian refugees on the labor market of the hosting countries in the early years of the crisis may differ from their impact in the following years. This can be caused by not only the large number of refugees in later years and the fact they became more geographically dispersed, but also attributed to the refugees' expectations about their future with the length of the crisis.

Jordan opened its doors to Syrian refugees since the beginning of the civil war. Since then, several studies have examined and reported the economic, political, social, and environmental impacts of Syrian refugees on Jordan (Lozi, 2013; Ajluni and Kawar 2014; Stave and Hillesund, 2015; Achilli, 2015; Turner, 2015; Fakih and Ibrahim, 2016; Alshoubaki and Harris, 2018, 2019; Alhawarin et al., 2018; Alougili, 2019; Aldayyat et al., 2019; Lenner and Turner, 2019; Harris, 2019; Salameh et al., 2020; Hussein et al., 2020; Mencutek and Nashwan, 2020; Al Wreikat and Al Kharabsheh, 2020). According to the United Nations High Commissioner for Refugees (UNHCR), Jordan is hosting 656,213 Syrian refugees registered with the UNHCR (as in April, 4th 2020). According to Jordan census data for 2015, the total number of Syrian refugees was 1,265,514 in 2015, of which approximately 83% are living in urban and rural areas outside of the refugee camps (Department of Statistics, 2019). Table 1 shows the geographical distribution of registered Syrian refugees with UNHCR in Jordan. It is not surprising that Amman is hosting nearly 30% of registered Syrian refugees since it is the center for economic activities in Jordan, and followed by the neighboring governorates to Syria such as Mafraq (24.7%), Irbid (20.6%, and Zarqa (14.5%), whereas the far governorates from the Jordanian border with Syrian received small number of refugees.

**Table 1:** Geographical distribution of registered Syrian refugees in Jordan (UNHCR).

Governorate	Population (UNHCR)	%
Amman	193,700	29.6%
Mafraq	162,228	24.7%
Irbid	134,803	20.6%
Zarqa	95,134	14.5%
Balqa	18,565	2.8%
Madaba	13,063	2.0%
Jarash	9,277	1.4%
Karak	8,453	1.3%
Maan	8,359	1.3%
Ajlun	6,537	1.0%
Aqaba	3,652	0.6%
Tafilah	1,716	0.3%

According MOPIC (2019), the direct cost of Syrian refugees on Jordan since 2011 is calculated to be USD 11,032 billion approximately. This total cost includes the costs of providing education, health, water, electricity, materials and goods, and other services to refugees as well as transport losses and security costs. In 2019, the total budget of Syrian refugees in Jordan equals USD 2.4 billion. Table 2 Shows the budget requirements of Syrian refugees in 2019 (MOPIC, 2019).

**Table 2:** Budget requirements of Syrian refugees in Jordan in 2019 (Million USD).

Sector	Cost
Education	220.5
Environment	3.1
Energy	26
Food Security	230
Health	213
Justice	17.6
Livelihoods	68.7
Local Governance and Municipal Services	61.3
Shelter	17.7
Social Protection	307
Transport	7.6
WASH Sector (Water, Sanitation and Hygiene)	229.1
Total for other: Subsidy, Security, Income Losses, Infrastructure Depreciation	998.2
Grand Total	2,400.2

Syrian refugees have affected Jordan’s economy, labor market and society through different aspects. The first effect was on the country’s finances. Although Jordan has weak economic growth, limited resources, and lack of public finances, it has provided temporary financial aid to Syrian refugees since 2011. According to MOPIC (2019), the direct and indirect cost of hosting Syrian refugees reached approximately $7.9 billion. Jordanian government pays additional cost to provide services such as education, health, security, electricity, water, food for refugees, which together increased total public expenditure (Alshoubaki and Harris, 2018, 2019; Zaytoun et al., 2017). Secondly, the impacts of Syrian refugees on labor market were analyzed by several studies as migration is theoretically considered to increase labor supply and reduces employment and wages. Stave and Hillesund (2015) concluded that Syrian refugees have competed with Jordanian labors especially in the low skilled and low wage jobs in the informal sector and reported high rate of unemployment because of the Syrian refugee crisis. In contrast, Fakih and Ibrahim (2016) found that Syrian refugees have no effects on the Jordanian labor market. Additionally, Fallah et al. (2019) also concluded that refugees have no negative effects on labor market at hosting regions. In this regards, Esen and Oğuş Binatlı (2017) stated that refugees did not have an obvious impact in the early years on labor markets of the hosting country. In addition, refugees normally start working in the informal sector, and the work in such jobs is normally illegal and does not require work permit. Before 2016, Syrians refugees were not officially allowed to work in Jordan as in other hosting countries such as Turkey. After 2016, Jordan received macro financial assistance, humanitarian aid, and trade concessions from the European Union and it allowed Syrian to access the labor market in return in some certain sectors such as agriculture, construction, and manufacturing (Fallah et al., 2019). According to Del Carpio and Wagner (2015), refugees represent a supply shock in the informal sector and a demand shock in the formal sector of the hosting country. Hence, refugees are anticipated to replace locals in the informal sector and low-wage jobs and decrease the average wage. Thirdly, the Syrian refugees' have also some effects on services. One of the most important aspects is the impacts of the massive influx of Syrian refugees on services in Jordan such as education, housing, water, energy, security, and infrastructure. The impacts on the educational sector can be seen from the increase in the number of students in the classroom, the increase of the students-teacher ratios, and reducing the duration of the school day following the double shifts policy employed in the governorates that have significant number of Syrian refugees (Assaad, 2018). With regard to housing sector, Syrian refugees have positively raised up housing rents especially in the hosted provinces due to the increased demand, which negatively affected locals who compete with refugees on housing (Assaad, 2018; Alhawarin et al., 2018; Tumen, 2016). In Turkey which has also received large number of Syrian refugees, several studies concluded that refugees affected the prices of goods and services negatively, and positively on housing rents (Tumen, 2016; Balkan and Tumen, 2016). On the other hand, Jordan is already listed as one of the most water scarcity regions in the world where local population struggle to access safe and adequate fresh water. With this massive influx of Syrian refugees, the country faces shortage in freshwater not only for refugees but also for its own inhabitants, which put more pressure on the limited water resources (Agrawal, 2018). Zaytoun et al. (2017) also found some other negative impacts of Syrian refugees on other sectors such as health, energy, security, and infrastructure. Fourthly, the Syrian refugees' influx has undoubtedly affected the age structure of the hosting countries' population such as Jordan and may influence the country’s demographic opportunity.

Syrian refugees in Jordan were primarily accommodated in refugee camps such as Za’tari and Azraq camps. Lately, they started to move and settle outside these camps looking for jobs and better life conditions, in the neighboring governorates such as Mafraq and Irbid as well as Amman being the capital and the main center of jobs and services in Jordan. Nowadays, it is clear that conflict in Syria will not end soon and the majority of Syrian refugees will stay in Jordan for a longer period than expected. Therefore, the current study focuses upon the way that Jordanians perceive Syrian refugees impacts on their country after nearly 10 years of the crisis. In particular, the study seeks to achieve the following objectives:

- Demonstrate the negative economic, social, and environmental impacts of Syrian refugees on Jordan from Jordanians point of view.
- Determine the positive impacts of Syrian refugees on Jordan according to Jordanians.
- Explore the perceptions of Jordanians toward Syrian refugees' crisis and their suggestions to deal with it in Future.

### 1.1 Data sources and methods

Required data of this research were obtained from different sources including secondary data about Syrians refugees' numbers and economic activities from Jordan’s Department of Statistics (DOS), Economic Research Forum (ERF), the Central Bank of Jordan, and UNHCR. In addition, an online survey was designed and distributed to Jordanians, through social media (Facebook), to collect the required primary data, where 1080 questionnaires were returned from respondents representing all governorates in Jordan and used in the analysis. The informed consent was obtained from all participants of this study. The instrument of the study consists of four parts including socio-economic characteristics of respondents; negative impacts of refugees; positive impacts of refugees; and suggestions to deal with the crisis. Five-Point-Likert scale was used for the last three parts in the questionnaire ranging from 1 (strongly disagree) to 5 (strongly agree). The Institutional Review Board/the Research Ethics Committee at the University of Jordan has reviewed and approved the study's objectives and procedures.

Descriptive statistics were used to analyze the obtained data using frequency analysis. Moreover, T-test and One-Way-ANOVA test were used to examine the study hypotheses using SPSS (V.23).

## 2 Results and discussion

### 2.1 Demographic statistics

A total of 1080 surveys were used in the analysis. The demographic characteristics of the respondents are described in Table 3. As seen in Table 3, the gender composition of respondents was relatively equal with 53.9% male respondents***.*** The dominant age group of respondents was below 25 years (59.3%), followed by 25–39 years (29.4%) due to the nature of the questionnaire being distributed online. The percentage of bachelor degree holders is (58.3%), as the dominant category, followed by postgraduates (22.8%), showing a high rate of educational level that Jordan is characterized by. Regarding monthly household income, the vast majority of respondents were characterized as low income receiving less than 800 JOD (62.1%), followed by the moderate income group who receive between JOD 800 and JOD 1599 (27.2%). Most of respondents (77.4%) live at the middle region and mainly in Amman (55.7%), followed by the northern region (20.4%).

**Table 3:** Respondents profile (N = 1080).

Variable	Respondents characteristics	Frequency	(%)
Gender	Male	582	53.9
	Female	498	46.1
Age (years)	Less than 25	640	59.3
	25–39	318	29.4
	40 and above	122	11.3
Educational level	Secondary and below	204	18.9
	Bachelor	630	58.3
	Post graduate	246	22.8
Family income (monthly JOD)	Less than 400	246	22.8
	400–799	424	39.3
	800–1199	176	16.3
	1200–1599	118	10.9
	1600–1999	32	3.0
	2000 and above	84	7.8
Place of Living	North region	220	20.4
	Middle region	836	77.4
	South region	24	2.2

### 2.2 Negative impacts of Syrian refugees in Jordan

Several pessimistic voices argues that refugees' impacts on host countries are mainly negative. The respondents of this study indicated that Syrian refugees have negative economic, social and environmental impacts on Jordan (Table 4). From the table it can be clearly seen that the study sample reported more negative economic impacts for refugees on Jordan (75.2%) comparing to their social impacts (68%) and environmental impacts (70.8%). According to respondents, the refugees have negative economic impacts include competing locals on the available job opportunities and increasing unemployment rates; increasing house rents, land and real-estate prices and goods' prices; and pressure on housing, limited natural resources, energy and other services. In this regard, Fakih and Ibrahim (2016) found no significant impacts for Syrian refugees on the labor market in Jordan in the early years of the crisis due to the fact that Syrian refugees were not allowed to work officially before 2016 in Jordan. In the early years of the crisis, the impacts of refugees were mainly on the informal sector. Syrians were allowed to work in Jordan after 2016 in some certain sectors and, thus, their impacts on the labor market were clearly significant in the recent years in both the formal and informal sectors (Fallah et al., 2019). Lozi (2013) stated that Syrian refugees' influx has led to an increase in the demand of food that led to an increase of food prices and inflation rate, and put more pressure on public services, which contributed to an increase in government expenditure as a result. Atiyat (2019) also provide an evidence for the impacts of refugees on local communities in Mafraq, Jordan. She concluded that local communities in Mafraq welcomed refugees, established good relationships with them, offered jobs for them at the beginning of their arrival. However, this situation did not last for long time as they the two groups stated to compete over jobs and services. Additionally, Alhawarin et al. (2018) stated that poor people were negatively affected by the increase in house rents and started competing with refugees over housing, in which the owners of such properties has experienced an increase of their revenues from housing. Mencutek and Nashwan (2020) and Alshoubaki and Harris (2018) also stated that Jordanians perceive refugees' employment in Jordan, as a source of competition for the already limited job opportunities.

**Table 4:** Negative impacts of Syrian refugees in Jordan from Jordanians' perspective.

Items	M	S.D	alpha
Economic impacts	3.76	.945	87
Competing locals on jobs	4.07	1.180	
Increasing house rents	4.01	1.147	
Pressure on housing	4.00	1.109	
Pressure on the limited national resources	3.83	1.221	
Negative effects on Jordanian labors	3.81	1.224	
Pressure on energy sector	3.74	1.192	
Increasing goods prices	3.70	1.246	
Pressure on security and defense services	3.63	1.252	
Negative effects on Jordanian national economy	3.56	1.187	
Increasing unemployment rates	3.56	1.293	
Increasing prices of lands and real-estates	3.49	1.294	
**Social impacts**	**3.40**	**1.039**	**.84**
Increasing crime rates	3.93	1.171	
Pressure on health services	3.82	1.168	
Pressure on educational services	3.74	1.208	
Negative impacts on Jordanian traditions	3.34	1.347	
Increasing drug abuse among youth	3.25	1.186	
Increasing harassments	3.22	1.335	
Increasing spinsterhood rates among Jordanian’s females	2.98	1.253	
Negative effects on life style and social relationships of Jordanians	2.90	1.322	
**Environmental Impacts**	**3.54**	**.989**	**.81**
Pressure on clean water	4.01	1.142	
Reducing water per capita in Jordan	4.00	1.059	
Pressure on infrastructure	3.89	1.127	
Groundwater pollution due to lack of sewage	3.54	1.260	
Air pollution	3.40	1.281	
increasing solid waste quantities	3.28	1.352	
Increasing noise rates	2.67	1.261	

Jordan Response Plan summarized the macroeconomic impact of the Syria refugees on Jordan using some key economic indicators in absolute and relative terms as follows (MOPIC, 2019):

- A decrease in GDP growth rate from 132% during the period 2004–2010 to 39% during 2011–2017, as the GDP growth declined by 70.5% after the crisis and that the trade balance deficit increased by 83%.
- An increase of unemployment rate from 12.5% in 2010 to 18.5% in 2017,
- An increase in public debt (national and foreign) by 103% during the period 2011–2017 compared to 59% during 2004–2010,
- A rise in the ratio of debt to GDP from 65% in 2011 to 96% in 2017,
- A decrease in the number of tourists (thousands for package) after 2011, and
- A decrease in the generated income growth from tourism to be 23% for the period 2011–2017 compared to 170% for the 2004–2010 period.

Secondly, the negative social impacts of refugees comprise increasing crime rates and drug abuse among youth; pressure on health and educational services; affecting Jordanian traditions, social relations and their life style; and increasing spinsterhood rates among Jordanian’s females. Accordingly, 82% of respondents believe that Syrian refugee’s crisis represents a real and complicated problem to Jordan, and 66.1% of respondents expect that this crisis and its circumstances will not end in the near future. Alshoubaki and Harris (2018) and Haynes (2016) reported some socio-cultural threats of Syrian refugees on Jordan such as changes in demographic structure, identity, and social habits and customs that led to social tension between the two groups. In addition, Alshoubaki and Harris (2018) discussed the role of refugees in the increase of criminal threats including crimes, smuggling, drug trafficking, and spillover of radicalism. Additionally, they claimed that there is a tension between host communities and Syrian refugees in Jordan due to the difference in both groups' habits and customs alongside with their bad living conditions. Atiyat (2018) also indicated that Jordanians and Syrians competed over public services such as health and education and infrastructure.

Finally, the main negative environmental impacts of refugees are summarized by pressure on clean water and infrastructure; ground water pollution; air pollution; increasing solid waste quantities and noise rates. From literature, Syrian refugees have contributed to increase the solid waste quantity by 340 tons daily across Jordan, which has burden the solid waste management sector especially in the main hosting cities such Mafraq, Irbid, Amman (Aldayyat et al., 2019; Saidan et al., 2017; Ministry of Municipal Affairs, 2017). Jordan is considered as a poor country with limited natural resources and suffers from a shortage in clean water and energy resources. Therefore, many negative environmental impacts of Syrian refugees in Jordan were indicated by previous studies including pressure on clean water, energy resources, and transportation (Farishta, 2014; Alshoubaki and Harris, 2018; Hussein et al., 2020; Al Wreikat and Al Kharabsheh, 2020). Additionally, they contributed to desertification, air pollution and solid waste (Aldayyat et al., 2019; Saidan et al., 2017).

### 2.3 Positive impacts of Syrian refugees in Jordan

Regardless the above-mentioned negative impacts of Syrian refugees, the respondents indicated some positive impacts of refugees. These positive impacts include providing the Jordanian market with well-trained, skilled, and low wage labours as well as creating more job opportunities to serve this large number of population as new demand for good and products. In addition, refugees with low wages increase the competitiveness of Jordanian products for the decrease in their cost and their prices as a result. This positive impact could be from employer’s perspective. However, this also can be perceived as a negative impact by the Jordanian workers. Moreover, more economic investments were created and international financial aid was provided for Jordan as well for hosting Syrian refugees. Respondents also believe that hosting refugees has contributed to position Jordan on the world map for its humanitarian role in providing shelter for those in need (See Table 5).

**Table 5:** Positive impacts of Syrian refugees in Jordan from Jordanians' perspective.

Items	M	S.D	alpha
Positive impacts	3.40	.623	.85
Providing Jordanian market with low wage labors	3.71	1.175	
Creating job opportunities to serve Syrian refugees	3.64	1.141	
Low wages increase competitiveness of Jordanian products	3.62	1.132	
New demand (market) for Jordanian goods and services	3.47	1.223	
Enhancing Jordan’s position in the international community for humanitarian coping with the Syrian refugees	3.38	1.216	
Additional well trained and skilled labors	3.34	1.210	
Low wages decrease products' prices	3.33	1.191	
Increasing self-steam and social ties with Syrians among Jordanians	3.30	1.140	
New economic investments	3.26	1.206	
Increasing financial support for Jordan from international community	2.90	1.226	

### 2.4 Suggestions to deal with Syrian refugees in Jordan

Refugees issue represents a very complicated problem in Jordan as the country are required to make a balance between its citizens need and refugees' need and between solidarity and hospitality. Respondents of the current study suggested some policies and solutions to deal with refugees' crisis in Jordan as summarized in Table 6. Firstly, respondents suggest building sewage networks for Syrian camps to overcome the pollution caused by these camps as well as targeting refugees in awareness campaigns for a wise water consumption. Secondly, they propose targeting Jordanians financially and in jobs by legislations especially in the most affected governorates. This can be achieved by giving the priority for Jordanians in the labor market and through large campaign of fund raising to support the affected governorates and local communities in generating new jobs and providing adequate services. Finally, the country should continue its vital role in seeking political solution to end this crisis in order to enable refugees to return home. Previous studies provided some suggestions to mitigate the impacts of Syrian refugees in Jordan such as the integration between public and private sectors in Jordan as such partnership will contribute to formulate long term and comprehensive response to this issue and it is considered as a crucial solution to complicated issues (Alshoubaki and Harris, 2018; Ostrom 1990). Atiyat (2018) suggested more international aids to Jordan, which support the country in providing the necessary need for both citizens and refugees. In addition, the study recommended Jordan to benefit from the financial aid by adopting clear actions of a wider plan in order to integrate Syrian refugees with local communities by creating jobs for both groups to alleviate potential tension between them.

**Table 6:** Suggestions to deal with Syrian refugees’ in Jordan from Jordanians perspective.

Items	M	S.D	Alpha
Suggestions to deal with Syrian refugees	3.83	.708	.83
Sewage network for Syrian camps to avoid negative environmental impacts	4.22	.987	
Increasing the of awareness toward water consumption	4.20	1.020	
Seeking political solution to end Syrian crisis	4.17	1.173	
Targeting Jordanians financially	4.07	1.151	
Giving priority to Jordanians in job opportunities by legislations	4.06	1.145	
Benefiting from Syrian businessmen' investments	3.95	1.237	
Supporting hosting communities of Syrians (affected governorates)	3.65	1.243	
Integrating Syrian refugees with Jordanian community	3.35	1.447	
Settling refugees outside crowded cities	2.81	1.540	
		

### 2.5 Gender effects on the perception of Jordanians toward Syrian refugees' impacts

Independent T-test results provided in Table 7, show that there are significant effects of respondents' gender on their perception toward Syrian refugees' negative impacts including economic, social and environmental impacts. This can be attributed to the higher unemployment rate among Jordanian females, and from another side because of the nature of women being more sensitive toward the social changes happened in Jordan after receiving refugees. In general, females gave more significance to the impacts of refugees than males. On the other hand, the results of T-test showed no significant effects of respondents' gender on their perceptions toward positive impacts of refugees.

**Table 7:** Independent T-test results for gender effects on the perception of Jordanians toward Syrian refugees' impacts.

Gender	DV	N	Mean	S.D	t	Df	Sig.2-tailed
Male	Negative economic impacts	582	3.70	.983	−2.201	1078	.028
Female	Negative economic impacts	498	3.88	.890			
Male	Negative social impacts	582	3.15	1.058	−2.778	1078	.006
Female	Negative social impacts	498	3.40	1.003			
Male	Negative environmental impacts	582	3.37	1.003	−2.643	1078	.008
Female	Negative environmental impacts	498	3.59	.960			
Male	Positive impacts	582	3.35	.533	−1.857	1078	.064
Female	Positive impacts	498	3.45	.711			

### 2.6 Age effects on the perception of Jordanians toward Syrian refugees' impacts

As shown in Table 8, the results of One-Way-ANOVA Analysis revealed that there are significant effects of respondents' age on their perception toward the negative impacts of Syrian refugees in Jordan. In addition, the results also showed no significant effects of the respondents' age on their perception toward the positive impacts of refugees. In more details, the older respondents showed more agreement on the negative economic, social and environmental impacts of refugees representing more concern and fear about these influences in the future.

**Table 8:** The results of One-Way-ANOVA results for age effects on the perception of Jordanians toward Syrian refugees' impacts.

IV		DV	N	Mean	S.D	Sum of Squares	df	Mean Square	F	Sig.
Age	<25	Negative economic impacts	640	3.67	.969	9.753	2	4.877	5.671	.004
	25–39	Negative economic impacts	318	3.93	.867					
	40+	Negative economic impacts	122	4.00	.942					
Age	<25	Negative social impacts	640	3.14	1.026	15.577	2	7.789	7.582	.001
	25–39	Negative social impacts	318	3.41	.973					
	40+	Negative social impacts	122	3.55	1.180					
Age	<25	Negative environmental impacts	640	3.34	.993	15.318	2	7.659	8.298	.000
	25–39	Negative environmental impacts	318	3.70	.956					
	40+	Negative environmental impacts	122	3.56	.944					
Age	<25	Positive impacts	640	3.37	.605	1.830	2	.915	2.369	.095
	25–39	Positive impacts	318	3.43	.664					
	40+	Positive impacts	122	3.40	.616					

### 2.7 Educational level effects on the perception of Jordanians toward Syrian refugees' impacts

Regarding educational level, the results of One-Way-ANOVA Analysis revealed that there are no significant effects of respondents' educational level on their perception toward the impacts of Syrian refugees in Jordan. These results indicate similar level of agreement on the refugees' impacts among respondents from different educational levels (Table 9).

**Table 9:** The results of One-Way-ANOVA results for educational level effects on the perception of Jordanians toward Syrian refugees' impacts.

IV		DV	N	Mean	S.D	Sum of Squares	df	Mean Square	F	Sig.
Educational level	Secondary school	Negative economic impacts	204	3.75	.945	.683	2	.341	.397	.673
	Bachelor	Negative economic impacts	630	3.73	.937					
	Post. Gard.	Negative economic impacts	246	3.97	.950					
Educational level	Secondary school	Negative social impacts	204	3.27	1.055	1.026	2	.513	.500	.607
	Bachelor	Negative social impacts	630	3.19	1.025					
	Post. Gard.	Negative social impacts	246	3.45	1.047					
Educational level	Secondary school	Negative environmental impacts	204	3.41	.982	1.156	2	.578	.626	.535
	Bachelor	Negative environmental impacts	630	3.41	1.003					
	Post. Gard.	Negative environmental impacts	246	3.69	.931					
Educational level	Secondary school	Positive impacts	204	3.38	.596	.610	2	.305	.789	.455
	Bachelor	Positive impacts	630	3.41	.588					
	Post. Gard.	Positive impacts	246	3.37	.730					

### 2.8 Income effects on the perception of Jordanians toward Syrian refugees' impacts

The results of One-Way-ANOVA Analysis revealed that there are no significant effects of respondents' monthly income levels on their perception toward refugees' impacts on Jordan except for environmental negative impacts (Table 10). In this regard, higher income group showed more concern toward refugees' negative environmental impacts.

**Table 10:** The results of One-Way-ANOVA results for monthly income effects on the perception of Jordanians toward Syrian refugees' impacts.

IV		DV	N	Mean	S.D	Sum of Squares	df	Mean Square	F	Sig.
Monthly income	<800	Negative economic impacts	670	3.79	.973	.573	2	.287	.320	.726
	800–1599	Negative economic impacts	294	3.74	.909					
	1600+	Negative economic impacts	116	3.86	.978					
Monthly income	<800	Negative social impacts	670	3.31	1.060	1.80	2	.901	.833	.435
	800–1599	Negative social impacts	294	3.18	.928					
	1600+	Negative social impacts	116	3.23	1.178					
Monthly income	<800	Negative environmental impacts	670	3.50	1.025	7.610	2	3.805	3.935	.020
	800–1599	Negative environmental impacts	294	3.31	.857					
	1600+	Negative environmental impacts	116	3.71	1.033					
Monthly income	<800	Positive impacts	670	3.43	.630	1.960	2	.980	2.537	.080
	800–1599	Positive impacts	294	3.30	.666					
	1600+	Positive impacts	116	3.45	.420					

## 3 Conclusion

The current study examined the impacts of Syrian refugees on Jordan from Jordanians' perspective. As Jordan is one of the countries that received large number of Syrian refugees starting from 2011 following the conflict in Syria, there were some noticed economic, social and environmental impacts on Jordan. The study shed the light on how these impacts were perceived by Jordanians after nearly one decade. According to the results of 1080 questionnaires, the respondents indicated high level of agreement upon the negative impacts of Syrian refugees on economic, social and environmental aspects in Jordan. Economic impacts were the most perceived impacts by respondents' who believe that Jordan was negatively affected by refugees in terms of pressure on labor market, housing, and services, alongside with the increase in the prices of goods, lands, and real-estates. These results are consistent with previous studies that examined the economic impacts of refugees (Fallah et al., 2019; Harris, 2019; Fakih and Ibrahim, 2016; Lozi, 2013). Moreover, negative social impacts of refugees were also mentioned including the increase in crime rates and drug abuse among youth, pressure on health and educational services, and impacts on Jordanian traditions and life style. Lozi (2013) also stated that Syrian refugees have political and social impacts on Jordan. In addition, the negative environmental impacts were pressure on clean water, air and ground water pollution, and increasing solid wastes.

In contrast, there are some positive impacts of Syrian refugees according to the study sample. Syrian refugees provided the market with low-wage and skilled labors who contribute to increase the competitiveness of Jordanian products. Additionally, this large number of population represents a new demand and market for Jordanian products that creates more jobs. Finally, Jordan has obtained a respect before the international community for hosting this large number of refugees despite its limited resources.

It is clear that Syrian refugees' crisis will not be ended in the near future and, thus, the hosting countries of refugees have to deal with this crisis’s circumstances and adopt policies that alleviate the negative impacts. Accordingly, respondents suggested some policies and strategies to deal with this issue. Among these policies, the government financial aid should target Jordanians and legislations should give priority for them especially in affected hosting governorates. Some solutions were also suggested to overcome the negative environmental impacts such as building sewage networks for camps and settling refugees outside crowded cities.

## Declarations

### Author contribution statement

Hamzah Khawaldah: Conceived and designed the experiments; Performed the experiments; Analyzed and interpreted the data; Contributed reagents, materials, analysis tools or data; Wrote the paper.

Nidal Alzboun: Analyzed and interpreted the data; Contributed reagents, materials, analysis tools or data; Wrote the paper.

### Funding statement

This work was supported by Deanship of Scientific Research at the University of Jordan, Amman, Jordan (Ref. 2110).

### Data availability statement

Data included in article/supp. material/referenced in article.

### Declaration of interest’s statement

The authors declare no conflict of interest.

### Additional information

No additional information is available for this paper.
